# Cognitive labor and the older learner: a feminist perspective on intellectual work in later life

**DOI:** 10.1093/geront/gnaf289

**Published:** 2025-12-06

**Authors:** Diana Amundsen

**Affiliations:** Human Development, School of Education, The University of Waikato, Bay of Plenty, Aotearoa New Zealand

**Keywords:** Cognitive labor, Feminist gerontology, Older learners

## Abstract

This conceptual article advances a feminist gerontological perspective on the intellectual and emotional labor of older adults—particularly women. Building on theories of invisible labor, care ethics, and adult learning, this article argues that unpaid, informal knowledge work (e.g., caregiving, mentoring, volunteering, community education) constitutes a form of cognitive labor that is persistently under-acknowledged in aging and education discourse. The analysis highlights the gendered dimensions of later-life learning, contending that older women act as vital yet unrecognized intellectual actors in family and community life. Through a critical synthesis of literature across gerontology, feminist theory, and adult education, this article proposes a new framework for recognizing and valuing intellectual contributions of older learners beyond formal institutions. By documenting this perspective, this article challenges deficit narratives of aging, stimulates dialog about the ethical and political stakes of recognizing cognitive labor, and identifies directions for future research and policy to advance gerontological scholarship.

This forum article addresses a notable gap in existing scholarship. While feminist labor theory has illuminated the invisibility of reproductive and domestic work, and gerontology has examined intersections of age and gender, little sustained attention has been paid to the cognitive dimensions of older adults’ unpaid intellectual work. This absence leaves policy and educational theory ill-equipped to recognize the ongoing contributions of older women as epistemic agents in later life.

From the outset, it is essential to locate this analysis within an intersectional framework. Age, gender, race, indigeneity, class, sexuality, disability, and migration status intersect to shape who performs cognitive labor, how it is valued, and whether it is visible at all. Indigenous women’s intellectual contributions, for example, have been central to cultural continuity yet remain marginalized in colonial and Western gerontological frameworks. Working-class older women often bear heavy caregiving and community burdens without institutional support, while migrant grandmothers sustain transnational family networks through logistical and emotional work.

In this article, the author proposes the concept of “cognitive labor” to reframe later-life learning and knowledge work. Building on feminist theories of labor, as well as feminist gerontology, and adult education, she argues that unpaid, informal intellectual work—such as caregiving, mentoring, and cultural transmission—should be recognized as a significant form of knowledge production. This article proceeds by first outlining theoretical foundations, then examining key sites of cognitive labor through vignettes, and proposing a conceptual framework for recognition. Lastly, implications are considered for research, policy, and practice. In doing so, this conceptualization of cognitive labor challenges deficit models of aging and affirms older women as vital intellectual contributors in family, community, and cultural life.

## Introducing cognitive labor in later life

As global populations age, societies are increasingly confronted with questions about the roles, capacities, and contributions of older adults. Discourses surrounding “successful aging” (Rowe & Kahn, 1987; Waddell et al., 2024), “active aging” and “productive aging” (Martinson & Halpern, 2011; Nakagawa et al., 2021; Rowe & Kahn, 1997) have gained traction in policy and academic arenas alike. Yet, these frameworks often focus on economic productivity or health maintenance, overlooking less visible but equally vital contributions (Koh et al., 2024)—particularly those involving unpaid intellectual and emotional work (Calasanti & Slevin, 2013; Charmes, 2019; Ervin et al., 2025).

At the same time, a renewed focus on lifelong learning reflects recognition of the cognitive and social benefits of continued engagement (Formosa, 2019; Narushima, 2008). However, dominant models of adult education remain narrowly focused on formal or credentialed learning, risking the ability to capture the full range of ways older adults—especially women—contribute knowledge, sustain social networks, and perform complex cognitive tasks in their everyday lives (Boulton-Lewis, 2010; Larri & Whitehouse, 2024; Withnall, 2010).

This article proposes the concept of cognitive labor as a way to reframe later-life learning and knowledge work. Building on feminist literature about invisible, emotional, and intellectual labor (Daniels, 1987; Federici, 2012; Hochschild, 2012), the author defines cognitive labor as: the unpaid, often informal, mental work involved in caregiving, mentoring, managing family systems, transmitting knowledge, and sustaining community life. For older adults, such labor includes navigating healthcare, guiding younger generations, facilitating community education, and offering ethical or cultural wisdom—forms of work often rendered invisible in both gerontological and educational discourse.

The concept of cognitive labor allows us to foreground the intellectual contributions made by older learners—particularly women—outside traditional institutions. Using a cognitive labor lens, the readers can examine the unseen thinking, strategizing, emotional intelligence, and ethical reasoning embedded in many caregiving and educational roles that older women routinely perform. These are not simply acts of compassion or service but are rich with cognitive complexity.

This article therefore argues for a feminist re-conceptualization of learning in later life—one that recognizes and values unpaid cognitive contributions. By reconceptualizing these activities as forms of intellectual work, this article aims to expand how we understand learning, teaching, and contribution in later life. Drawing together insights from feminist theories of labor, feminist gerontology, and adult education, the author would like to propose a new conceptual framework that recognizes older adults not merely as learners, but as knowledge workers engaged in continuous (often under-acknowledged) intellectual production. In doing so, this suggested new framework explicitly recognizes unpaid educational labor in later life, centers the experiences of older women, and, draws from feminist critiques of work and aging.

## Theoretical foundations

### Conceptual underpinnings

The concept of cognitive labor is used here to denote the intellectual, emotional, and reflective work through which older adults—particularly women—generate, sustain, and share knowledge. While the term has appeared in sociological and feminist scholarship to describe the mental and affective effort embedded in everyday life (Daminger, 2019), this article extends the concept into later-life learning contexts. Cognitive labor in later life encompasses acts of mentoring, storytelling, writing, reading, and problem-solving that contribute to personal meaning-making and to collective cultural continuity.

From a feminist gerontological perspective, cognitive labor offers a way to conceptualize aging not as decline but as ongoing epistemic participation. This framing challenges narrow biomedical narratives of cognitive aging by emphasizing the relational and socially embedded nature of intellectual work. It positions older women as active knowledge producers whose unpaid cognitive contributions enrich families, communities, and civic life.

To situate this argument within a global context, the paper references aligned international research that underscores how cognitive and intellectual contributions in later life are recognized across diverse settings. Illustrative examples include studies from China (Yu & Barr, 2025), Canada (Charpentier & Kirouac, 2022), the United Kingdom (Formosa, 2021), Ethiopia, India, Mexico, Nepal, and Peru (Aikman & Robinson-Pant, 2019), as well as among older Hispanic adults (Yahirun et al., 2025). Together, these studies highlight cross-cultural expressions of cognitive labor and reaffirm the global salience of feminist perspectives on work, care, and lifelong learning in later life.

### Feminist theory

Feminist theory provides the conceptual grounding for this article, framing older women’s intellectual and cognitive labor as a socially situated and relational form of work. The analysis is positioned primarily within feminist standpoint epistemology, which asserts that women’s lived experiences—particularly those historically marginalized by age and gender—constitute a legitimate and generative source of knowledge (Harding, 1991). Within this perspective, the intellectual contributions of older women are not ancillary to formal productivity but represent crucial forms of epistemic and social participation (Huo & Kim, 2025; Yu & Barr, 2025).

At the same time, this article acknowledges insights from socialist and postmodern feminist traditions, which together draw attention to the structural and discursive conditions that shape women’s lives in later adulthood. Socialist feminism highlights how unpaid and often invisible forms of cognitive and emotional work sustain social systems (Arber et al., 2003), while postmodern feminism problematizes the fixed categories of “productive” and “retired” subjectivities (Calasanti & Slevin, 2013). These currents inform the interpretation of cognitive labor as historically contingent, relationally embedded, and culturally mediated.

This integrated framework extends feminist gerontology by foregrounding cognitive labor as an enduring site of agency and identity-making in later life. It underscores that intellectual work undertaken by older women—through teaching, mentoring, storytelling, and reflective inquiry—constitutes an important contribution to social knowledge production and cultural continuity.

### Feminist theories of labor

Feminist political economy has long exposed the structural devaluation of women’s unpaid work. In their grassroots International Wages for Housework Campaign for recognition and payment for caring work inside and outside the home, Mariarosa Dalla Costa (1972/1999) first highlighted how domestic and reproductive labor underpins capitalist economies. Later, Arlene Daniels (1987) and Arlie Hochschild (2012) showed how invisible and emotional labor sustain families and communities. More recently, Allison Daminger (2019) identifies the intellectual labor component of housework, conceptualizing the mental load of anticipating, planning, decision-making, and delegating tasks—cognitive processes that persist well into later life.

Often, the dynamics identified in feminist theories of labor are carried forward into older adulthood, where older women continue to shoulder much of the invisible, unpaid work of coordinating kin networks, caregiving, and community organizing. This is not only physical or emotional labor but, as Tronto (1993) reminds us, a deeply cognitive practice involving moral reasoning, judgment, and interpretation. In this sense, the mental load described by Daminger (2019) does not disappear with age but shifts into new domains of responsibility, sustaining families and communities across generations. Yet, feminist political economy highlights how this labor remains feminized, informal, and relational, and is therefore systematically excluded from economic measures and policy recognition (Fraser, 2016; Vogel, 2013).

### Feminist gerontology and recognition theory

Feminist gerontology builds on these feminized theories of labor by rejecting deficit-based views of aging and foregrounding older women as active contributors (Calasanti & Slevin, 2013; Daminger, 2019). Yet androcentric and neoliberal frameworks still marginalize older women’s roles, framing them as dependent rather than as experts with embodied knowledge (Arber et al., 2003)—an issue highlighted in intersectional analyses showing how gendered ageism plus ethnicity, disability, or sexuality exacerbate invisibility (Llurda-Marí, 2025; Merodio et al., 2024).

Andocentric and neoliberal frameworks cast older women as burdens rather than sources of support (Amundsen, 2022; Katz, 2002), especially in non-Western or disabled communities where their caregiving and community leadership are undervalued (Beban et al., 2025). Further, these frameworks are associated with stereotyping older women as frail rather than capable and dismissing their resilience, wisdom, and moral reasoning in policy, health, and social narratives (Beban et al., 2025; Merodio et al., 2024; Katz, 2019).

Recognition theory (Fraser, 1995; Honneth, 1995), thus, is a useful theoretical lens to understand how older women’s intellectual labor is routinely misrecognized. In recognition theory, symbolic erasure occurs when women’s contributions are absent from discourse, and material erasure occurs when policy provides no support for informal learning or caregiving. These dynamics intersect with colonial epistemologies that privilege credentialed, individual knowledge over oral, collective, and relational forms of knowledge.

Extending these insights even further, Indigenous feminist scholars (Goodyear-Ka‘ōpua, 2013; Simpson, 2017; Smith, 2012) show how Indigenous elder women sustain communities through ceremony, storytelling, and intergenerational teaching. These forms of cognitive labor are essential for cultural survival yet rarely valued in Western frameworks. Aotearoa New Zealand Māori *kuia* (grandmother, female elder), Aboriginal Elders in Australia, and Sámi women in northern Europe exemplify knowledge guardianship that combines pedagogy, ethical judgment, and cultural stewardship (Archibald, 2008; Bessarab, 2008). Recognition theory therefore opens a pathway toward revaluing feminized knowledge practices and preparing the ground for feminist and Indigenous perspectives that highlight alternative modes of intellectual labor.

### Adult education perspectives

Adult education scholarship extends recognition theory insights by showing how older women’s information teaching and learning are consistently undervalued within mainstream frameworks (Amundsen, 2020). For instance, Fricker’s (2007) notion of epistemic injustice captures the credibility deficits attached to their caregiving, mentoring, and storytelling when compared to credentialed forms of expertise. Feminist adult education theorists (Butterwick & Roy, 2016; Manicom & Walters, 2012; Tisdell, 1998) emphasize the centrality of identity, embodiment, and narrative—domains where older women frequently excel and innovate.

Approaching adult education through feminist frames validates peer-to-peer and intergenerational learning, which mainstream frameworks often dismiss. Wallace et al. (2025) have developed a feminist-critical gerontological framework for Black women (which aligns with this article’s feminist perspective on older women’s intellectual contributions). Indigenous pedagogies deepen this perspective still further, grounding education in reciprocity, land-based knowledge, and storytelling. First Nations, Sámi, and Inuit elder women preserve language, ecological wisdom, and cultural resilience through relational and dialogic teaching (Archibald, 2008; Caughey et al., 2024; Simpson, 2017). Such contributions resist Western assumptions of individualized achievement and reposition older women as cultural educators whose labor sustains continuity, resilience, and survival.

### Theoretical convergence

Taken together, feminist labor theory, recognition theory, and adult education perspectives provide a conceptual toolkit for reframing older adults’ unpaid intellectual work as cognitive labor. These theories expose how systemic undervaluation, epistemic injustice, and colonial hierarchies obscure the intellectual contributions of older women, while also pointing to alternative pedagogies that affirm them as educators, mentors, and cultural leaders. Grounded in these theories, the readers can now turn to analyze distinct sites where cognitive labor unfolds in later life.

## Sites of cognitive labor in later life

Cognitive labor in later life is performed across informal settings that are frequently overlooked by formal systems of recognition. Through caregiving, volunteering, and intergenerational knowledge transfer, older women engage in complex tasks involving memory, moral judgment, planning, negotiation, and teaching—hallmarks of intellectual work. The following three vignettes illustrate how this cognitive labor sustains families: communities, cultural continuity, and collective futures (see online supplementary material, Appendix 1 for more vignettes).

### Caregiving and kin-work

Vignette: Ellen in dementia care and ethical decision-making Ellen, a 78-year-old retired teacher, cares for her husband with advanced dementia. Each day she manages medications, monitors side effects, schedules health visits, and anticipates changes in behavior. She draws on years of observation to de-escalate agitation, balancing her husband’s autonomy with his safety, while coordinating with doctors and lawyers on care decisions. Ellen’s work requires judgment, memory, and ethical reasoning—revealing caregiving as an intellectual and moral project, not simply a physical one.

Other forms of caregiving and kin-work might include transnational caregiving, where grandmothers arrange monetary contributions and household routines across borders, or female elders in Indigenous communities who combine childcare with teaching genealogy and cultural customs. Across these contexts, caregiving entails coordination, interpretation, advocacy, and pedagogy. As Tronto (1993) observes, such care-based work involves attentiveness, responsibility, and moral reasoning—hallmarks of cognitive labor.

### Volunteering and community education

Vignette: Mere in a land protection group

Mere, a 72-year-old Indigenous older adult, chairs a regional land protection meeting. She cross-references legal documents with oral histories, ensuring ancestral narratives inform contemporary land claims, while also coordinating younger activists in outreach and protest logistics. Her labor is cognitive and political: interpreting legal jargon, recalling precedents, negotiating alliances, and teaching the next generation how to advocate.

Other examples of volunteering and community education might include retired nurses leading parenting classes, or older women facilitating digital literacy workshops in libraries. These roles demand strategic thinking, public speaking, and relational pedagogy. Yet they are often framed merely as “volunteering,” obscuring the intellectual depth of the work. Recognizing these contributions challenges ageist assumptions of decline and affirms older adults as active knowledge producers and civic leaders.

### Peer-to-peer and intergenerational knowledge transfer

Vignette: Mary uses language revival in the home

Mary, a 69-year-old First Nations grandmother, teaches her grandchildren their ancestral language through daily routines, songs, and stories. She collaborates with schools by sharing recordings, carefully discerning which narratives can circulate outside sacred contexts. This labor demands memory work, pedagogical skill, and ethical judgment. It is at once cognitive, relational, and political—sustaining language and resisting colonial erasure.

Comparable forms of peer-to-peer and intergenerational teaching might include mentoring grandchildren in digital authorship or leading storytelling circles that archive community histories. Such exchanges are dialogic and reciprocal, blending memory, critical reflection, and emotional intelligence. Naming them as cognitive labor affirms older women as epistemic authorities whose contributions shape both family life and collective futures.

### Connecting the sites of cognitive labor

Across caregiving, community leadership, and intergenerational teaching, older women enact unpaid intellectual labor that sustains social life yet remains under-acknowledged. These practices exemplify what Fricker (2007) calls epistemic injustice: Despite clear cognitive skill, their contributions are diminished because they occur outside credentialed or economic frameworks. Recognizing them as cognitive labor reframes later life as a site of active knowledge production and social continuity.

## Conceptual framework: recognizing cognitive labor

This section proposes a conceptual framework for understanding the cognitive labor of older adults as a form of unpaid intellectual work embedded in social, relational, and educational contexts. Drawing from feminist theories of labor, recognition theory, and adult education, the model makes explicit the links between informal learning, cognitive output, and social value. At its core is the argument that the mental, emotional, and ethical efforts older adults invest in caregiving, teaching, and mentoring deserve recognition as legitimate and valuable forms of knowledge production.

### The framework explained

**Figure 1.**
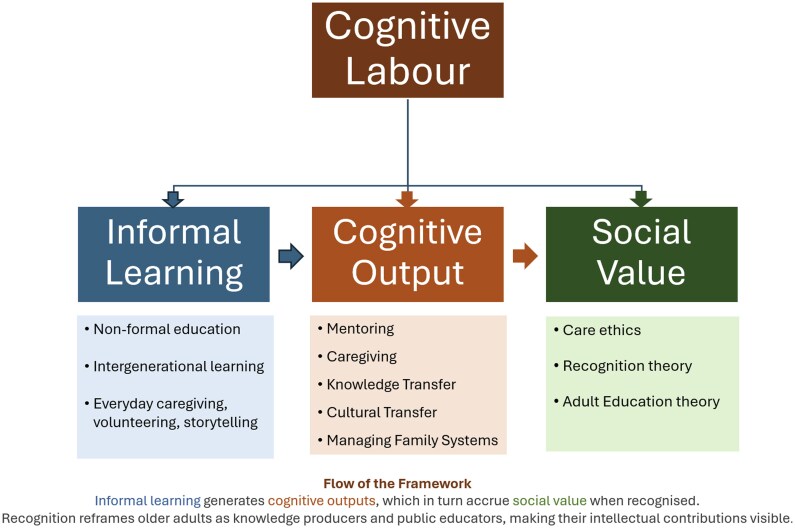
Recognizing cognitive labor.

The framework is organized around three interconnected domains which are more fully explained here and depicted summarily in Figure 1.

#### Informal learning

Nonformal education, along with intergenerational learning beyond institutional settings, forms part of this picture. Informal learning is embedded in everyday life—through caregiving, volunteering, and storytelling—and often driven by necessity, care, or community need rather than formal curricula. As Merriam and Bierema (2013) argue, much adult learning is experiential and socially constructed. In later life, informal learning typically moves beyond acquiring new knowledge toward the adaptive application of accumulated life experience. Within the proposed framework, this dimension recognizes that older adults are not just recipients of education but active learners and educators.

#### Cognitive output

At the heart of the model is the concept of cognitive labor—the intellectual and emotional effort involved in tasks such as mentoring, caregiving, managing family systems, transmitting knowledge (including cultural knowledge transfer), and sustaining community life. This labor is generative: It produces insight, guidance, social cohesion, and learning. Cognitive output, thus, is understood not simply as mental activity, but as relational intellectual work—a hybrid of memory, reasoning, emotional intelligence, and ethical reflection. Reframing these types of cognitive outputs helps expose the ways in which cognitive labor is routinely performed in unpaid and informal contexts, particularly by older women, without acknowledgement of its intellectual depth or social function.

#### Social value

The third dimension emphasizes the societal and ethical implications of recognizing this labor. Drawing on care ethics (Tronto, 1993), recognition theory (Fraser, 1995; Honneth, 1995), and adult education theory, this element of the framework situates cognitive labor within broader questions of justice, dignity, and inclusion. In particular, recognition theory provides a lens to understand how the social invisibility of older adults’ intellectual contributions is a form of misrecognition—a denial of their value as epistemic agents. Recognizing cognitive labor is thus not only a conceptual shift, but a political and pedagogical imperative.

These three domains—informal learning, cognitive output, and social value—are interdependent. Informal learning practices both generate and are sustained by cognitive labor. Cognitive outputs are shaped by the contexts in which older adults live and contribute, and these outputs accrue social value only when recognized by others. The interplay of informal learning, cognitive output, and social value highlights how social structures—such as ageism, sexism, and neoliberal individualism—often obscure or devalue the labor of older adults.

The model also reflects feminist commitments to relationality, context, and care. Older adults are positioned as knowledge producers and public educators—even when their work takes place in domestic or community settings. Not only does this model invite a conceptual shift in how learning and labor are theorized, but it also paves the way for thinking afresh about research and policy. Moving away from the formal and commodified, toward the collective, the intergenerational, and the ethically grounded, this model makes intellectual contributions of older adults visible and recognizable. By foregrounding recognition, the readers’ attention turns toward the urgent need for new research metrics, policy paradigms, and programmatic designs that make visible—and valuable—the cognitive labor of older learners.

## Implications

Research on aging and education has traditionally prioritized measurable outcomes such as participation rates, cognitive decline trajectories, or the economic valuation of volunteering. These approaches, while useful, miss the relational and affective dimensions of older adults’ intellectual work. Future scholarship should therefore broaden its scope to include unpaid cognitive outputs such as mentoring, cultural transmission, ethical guidance, and care coordination. Qualitative and participatory methodologies—such as story circles, yarning, or cognitive mapping—are particularly well suited to feminist research approaches, allowing older women’s own accounts of their intellectual labor to surface. By integrating feminist labor theory, recognition theory, and adult education, gerontological research can generate more nuanced understandings of how race, class, indigeneity, and disability shape whose contributions are visible and whose remain overlooked.

Policy frameworks also require significant rethinking. At present, most aging policies emphasize financial independence, healthcare access, and service delivery, with little attention to the unpaid intellectual and emotional work that sustains families and communities. This absence reflects an underlying assumption that older adults are primarily service *users* rather than *contributors*. A feminist recognition of cognitive labor has potential to shift this orientation by affirming older adults as knowledge holders and educators. Practical initiatives could include stipends or tax credits for unpaid carers and educators, expanded funding for intergenerational programs, and the incorporation of cognitive and emotional labor metrics into national aging and education strategies. For example, Gaugler et al. (2025) advocate building a public health infrastructure to support family caregivers of people with dementia—such ideas connect this discussion of intellectual and emotional labor with broader caregiving and aging-infrastructure discourses. Taking these measures would not only acknowledge the intellectual contributions of older adults but also embed them in policy systems that currently render their work invisible.

For practitioners, the implications are equally significant. Professionals in gerontology, social work, community development, and education can play a pivotal role in recognizing and supporting cognitive labor. Supporting cognitive labor involves designing programs that center lived experience by engaging older adults as co-teachers, mentors, and peer facilitators, rather than treating them solely as learners or recipients of care. Training for practitioners should include tools for identifying and affirming cognitive labor, particularly in caregiving and community contexts where such contributions are easily overlooked. Building feminist pedagogies that foreground storytelling, ethics, and reciprocal learning requires a conscious move away from deficit-based models of aging toward environments that validate and share older adults’ intellectual contributions.

Educational curricula also offer a powerful site for change. Community and tertiary programs can be reimagined to include co-designed courses led by elder women, or intergenerational storytelling projects, or land-based learning initiatives with cultural custodians. Embedding recognition of cognitive labor into curricula not only enriches educational content but also disrupts ageist and sexist assumptions about who counts as a teacher or a knowledge producer. Aligning these efforts with global initiatives such as the World Health Organization’s Decade of Healthy Aging (WHO, 2020, 2022) underscores the urgency of positioning older adults as co-creators of knowledge rather than passive recipients of services.

Recognizing cognitive labor as a vital component of older adults’ contributions—especially in unpaid and informal domains—therefore carries wide-ranging implications. Recognizing cognitive labor challenges the limitations of existing gerontological and educational frameworks, compels policymakers to account for forms of labor they have long ignored, and calls practitioners to reshape pedagogy and program design. Recognizing cognitive labor has the likely potential to affirm older women as intellectual agents whose work sustains families, communities, and cultures, and whose contributions deserve visibility and value within both scholarship and practice.

## Conclusion

This article has argued for a feminist re-conceptualization of later-life learning by introducing cognitive labor as a form of unpaid intellectual work central to aging societies. Undervaluing older women’s cognitive labor has profound political and social consequences. Misrecognition not only denies dignity to those performing this intellectual work in later life, but also erodes collective benefit by obscuring their contributions to family, community, and cultural continuity.

By tracing cognitive labor across sites such as kin-work, community education, and intergenerational knowledge transfer, the concept itself challenges deficit narratives that equate aging with decline or dependency. Instead, cognitive labor highlights later life as a period of sustained intellectual activity, relational learning, and cultural transmission. The conceptual framework proposed here invites researchers, policymakers, and practitioners to rethink what counts as learning, who counts as an educator, and how intellectual work is recognized.

Reframing aging as a time of active knowledge production is not merely an academic exercise but an ethical and political imperative. Inclusive lifelong learning systems and age-friendly societies require us to see, support, and value the intellectual contributions of older women, whose work continues to sustain communities and cultural survival.

I therefore close not with an answer, but with an invitation: that scholars and practitioners engage with, critique, and extend this model—whether by contesting its premises, testing its applicability across diverse contexts, or expanding its scope to include new forms of intellectual practice. In doing so, the framework may evolve as a collective endeavor, enriched through dialog and collaboration across disciplines, generations, and communities. What might a society look like if older women’s knowledge were placed at its center?

## Supplementary Material

gnaf289_Supplementary_Data

## Data Availability

This is a conceptual forum article and as such, no data were collected for analysis, other than the literature which has been cited within the Reference List of this article.

## Positionality statement

As an older Pākehā feminist scholar based in Aotearoa New Zealand, the author’s perspective is shaped by the responsibilities and privileges of working within a settler-colonial context. The author writes from within the field of adult education, informed by feminist theory, critical gerontology, and a commitment to social justice in later life. Her intellectual interests are rooted in community engagement, intergenerational learning, and the often-overlooked knowledge carried by older women in family and community life. While this article draws on both global and local sources, it is particularly shaped by her work alongside Māori colleagues and communities, whose contributions to education, caregiving, and knowledge transmission have informed her understanding of relational learning and cognitive labor.
