# Supplementary material for: Cognitive labor and the older learner: a feminist perspective on intellectual work in later life
Source: Gerontologist. 2025 Dec 6;66(1):gnaf289. doi: 10.1093/geront/gnaf289 (PMC12848226; doi:10.1093/geront/gnaf289)
Supplement: gnaf289_Supplementary_Data [file gnaf289_supplementary_data.pdf]

Cognitive Labor and the Older Learner: A Feminist Perspective on Intellectual Work in Later Life

Diana Amundsen, PhD[1]*

[1]Human Development, School of Education, The University of Waikato, Bay of Plenty, Aotearoa New Zealand

*Address correspondence to: Diana Amundsen, PhD. Email: diana.amundsen@waikato.ac.nz

Abstract

This conceptual article advances a feminist gerontological perspective on the intellectual and emotional labor of older adults—particularly women. Building on theories of invisible labor, care ethics, and adult learning, the paper argues that unpaid, informal knowledge work (e.g., caregiving, mentoring, volunteering, community education) constitutes a form of cognitive labor that is persistently under-acknowledged in aging and education discourse. The analysis highlights the gendered dimensions of later-life learning, contending that older women act as vital yet unrecognized intellectual actors in family and community life. Through a critical synthesis of literature across gerontology, feminist theory, and adult education, the article proposes a new framework for recognizing and valuing intellectual contributions of older learners beyond formal institutions. By documenting this perspective, the paper challenges deficit narratives of aging, stimulates dialog about the ethical and political stakes of recognizing cognitive labor, and identifies directions for future research and policy to advance gerontological scholarship.

Keywords: cognitive labor; feminist gerontology; older learners

Appendix 1: Illustrative Vignettes of Cognitive Labor in Later Life

The following vignettes extend the examples discussed within the main text. Each illustrates how older women enact unpaid intellectual and emotional work that sustains families, communities, and cultural continuity. Spanning diverse cultural settings, these brief portraits exemplify cognitive labor as an intersection of ethical reasoning, relational pedagogy, and social contribution.

**1. USA**<br>**Lorna navigating digital learning spaces**	**2. Aotearoa New Zealand**<br>**Aroha mentoring young mothers**
Lorna, a 68-year-old retiree and former librarian, volunteers at a local library's "Tech for Seniors" program. She helps peers and recent migrants navigate online services, social media, and digital privacy. Each session requires diagnostic thinking, adapting explanations to different literacy levels, and emotional sensitivity to learners' embarrassment or frustration. Lorna's ability to translate complex digital concepts into accessible language exemplifies how unpaid intellectual labor contributes to digital inclusion and civic participation.	Aroha, a 74-year-old Māori kuia, facilitates weekly gatherings at her marae for young mothers. Through stories about childbirth, ancestral women, and healing plants, she transmits intergenerational knowledge and moral guidance. Her mentorship involves discerning how much sacred knowledge to share, adapting narratives to the group's learning needs, and mediating conflict with humour and empathy. Aroha's storytelling is an act of pedagogy and cultural continuity—a cognitive and ethical labor that sustains both family and tribal well-being.
**3. Australia**<br>**Sushila coordinating interfaith dialogues**	**4. Canada**<br>**Denise documenting local history**
Sushila, a 70-year-old Indian Australian, coordinates an interfaith women's dialogue circle that meets monthly. She manages correspondence, selects readings, and moderates conversations about shared values and cultural practices. Her work requires analytical comparison, emotional diplomacy, and reflective listening across faith traditions. By facilitating mutual understanding, Sushila enacts cognitive labor that bridges generational and cultural divides while fostering community cohesion.	Denise, a 76-year-old widow living in rural Canada, leads a small group of older women compiling a digital archive of community photographs and oral histories. She curates sources, verifies dates, and writes narrative captions linking personal memory to regional events. Her archival labor is historical, interpretive, and ethical—deciding how to represent others' voices respectfully while preserving collective memory. Through this unpaid scholarship, Denise transforms everyday recollection into public knowledge.
**5. United Kingdom**<br>**Grace mentoring about climate-action**	**6. Mexico**<br>**Marisol facilitating memory circles**
Grace, a 67-year-old environmental activist in the United Kingdom, mentors younger members of a community climate-action collective. She teaches grant-writing, public speaking, and consensus-building, drawing on her prior experience as a teacher. Grace's mentoring requires rhetorical precision, strategic planning, and moral discernment about advocacy tactics. Her guidance embodies cognitive labor that sustains intergenerational activism and situates older	Marisol, a 71-year-old retired teacher in Oaxaca, coordinates a círculo de memoria—a neighborhood storytelling group where elders share recollections of local traditions, recipes, and resistance movements. She organizes sessions, records oral histories, and trains younger volunteers in interviewing techniques. Marisol's labor involves translation between Spanish and Zapotec, ethical decisions about what stories to publish, and curatorial thinking about how community

women as intellectual anchors in social movements.	memory should be represented. Her cognitive labor sustains cultural identity while positioning older women as public historians and teachers.
**7. China**<br>**Liling guiding community learning**	**8. Nepal**<br>**Tara curating women's life stories**
Liling, a 69-year-old former accountant from Chengdu, leads a volunteer literacy group for older migrant women in her housing complex. Each week she designs short lessons that integrate reading with everyday problem-solving—bus schedules, medicine labels, online payments. Beyond literacy instruction, she facilitates conversations about family, migration, and self-worth, prompting participants to connect practical tasks to their own life stories. Liling's planning, linguistic dexterity, and emotional attunement reveal the intellectual and relational dimensions of unpaid teaching in later life.	Tara, a 73-year-old grandmother and former midwife in Kathmandu, collaborates with a local NGO to document women's reproductive health experiences across generations. She conducts interviews, verifies timelines, and helps translate narratives into Nepali and English for publication. The work requires critical listening, medical knowledge, and negotiation with community leaders about consent and representation. Through this participatory documentation, Tara performs cognitive labor that transforms lived experience into shared public knowledge, bridging oral tradition and feminist research practice.

Concluding Note

Taken together, these vignettes highlight the multiplicity of cognitive labor enacted by older women across cultural contexts. Whether expressed through caregiving, teaching, storytelling, activism, or archiving, each account illustrates how unpaid intellectual work contributes to social cohesion and intergenerational learning. Recognizing such practices as cognitive labor challenges deficit narratives of aging and affirms older women as vital knowledge producers whose intellectual contributions sustain communities worldwide.
